# Supplementary material for: Thinned Nectarines, an Agro-Food Waste with Antidiabetic Potential: HPLC-HESI-MS/MS Phenolic Characterization and In Vitro Evaluation of Their Beneficial Activities
Source: Foods. 2022 Mar 30;11(7):1010. doi: 10.3390/foods11071010 (PMC8997825; doi:10.3390/foods11071010)
Supplement: Supplementary file 1 [file foods-11-01010-s001.zip › foods-1646081-Supplementary.pdf]

SUPPLEMENTARY MATERIALS

Table S1. Linearity range, limit of detection (LOD) and limit of quantification (LOQ) for polyphenols analysis.

Compound	Linearity range (ppm)	Calibration curve	LOD (ppm)	LOQ (ppm)
Gallic acid	0.1 – 1000	y = 2E+07x – 28492	6.240	18.908
Chlorogenic acid	0.1 – 1000	y = 3E+07x – 30148	7.102	21.522
Catechin	0.1 – 1000	y = 9E+06x – 11907	5.405	16.377
Epicatechin	0.1 – 1000	y = 1E+07x – 4563.5	4.849	14.695
Procyanidin B1	0.5 – 1000	y = 3E+06x – 3730.6	5.518	16.722
Procyanidin B2	0.5 – 1000	y = 8E+06x – 8832.4	5.200	15.758
Rutin	0.1 – 1000	y = 2E+07x – 1076.9	2.372	7.189
Quercetin	0.1 – 1000	y = 5E+07x – 41742	2.893	8.766

Table S2. Intra- and inter-day precision for polyphenols analysis.

Analyte	Concentration (ppm)	Precision	
		Intra-day (%, *n*=3)	Inter-day (%, *n*=3)
Gallic acid	0.1	2.620	3.172
	0.05	1.682	1.300
	0.01	0.896	0.547
Chlorogenic acid	0.1	0.657	1.491
	0.05	2.285	2.931
	0.01	2.952	4.876
Catechin	0.1	0.611	0.122
	0.05	1.463	1.867
	0.01	8.860	7.974
Epicatechin	0.1	2.350	2.296
	0.05	7.913	1.800
	0.01	7.613	7.319
Procyanidin B1	0.1	1.539	8.147
	0.05	0.995	1.105
	0.01	2.202	2.312

Procyanidin B2	0.1	0.819	2.663
	0.05	1.328	1.949
	0.01	1.258	3.536
Rutin	0.1	1.354	1.378
	0.05	0.126	2.913
	0.01	1.922	2.920
Quercetin	0.1	0.360	4.637
	0.05	1.375	6.975
	0.01	2.276	4.260

Table S3. Intra- and inter-day accuracy for polyphenols analysis.

Analyte	Concentration (ppm)	Precision	
		Intra-day (%, *n*=3)	Inter-day (%, *n*=3)
Gallic acid	0.1	3.508	3.926
	0.05	1.272	1.506
	0.01	0.368	0.394
Chlorogenic acid	0.1	-2.960	-2.980
	0.05	-1.483	-1.574
	0.01	-0.184	-0.196
Catechin	0.1	1.312	1.284
	0.05	0.076	-0.080
	0.01	-0.564	-0.509
Epicatechin	0.1	0.894	0.882
	0.05	0.620	1.250
	0.01	-0.011	0.144
Procyanidin B1	0.1	-0,507	-1,075
	0.05	-0,488	-0,486
	0.01	-0,189	-0,192
Procyanidin B2	0.1	-0.612	0.814
	0.05	-0.390	0.007
	0.01	-0.122	0.008

	0.1	0.601	0.643
Rutin	0.05	0.100	0.001
	0.01	0.034	0.020
	0.1	-2.765	-2.788
Quercetin	0.05	-1.561	-1.709
	0.01	-0.261	-0.265
